# Alpha-1-antitrypsin as novel substrate for *S. aureus’* Spl proteases – implications for virulence

**DOI:** 10.3389/fimmu.2024.1481181

**Published:** 2024-11-19

**Authors:** Franziska Scherr, Murthy N. Darisipudi, Friedemann R. Börner, Sophie Austermeier, Franziska Hoffmann, Martin Eberhardt, Goran Abdurrahman, Christopher Saade, Ferdinand von Eggeling, Lydia Kasper, Silva Holtfreter, Barbara M. Bröker, Michael Kiehntopf

**Affiliations:** ^1^ Institute of Clinical Chemistry and Laboratory Diagnostics, Jena University Hospital, Jena, Germany; ^2^ Institute of Immunology, University Medicine Greifswald, Greifswald, Germany; ^3^ Department of Microbial Pathogenicity Mechanisms, Leibniz Institute for Natural Product Research and Infection Biology -Hans Knoell Institute Jena (HKI), Jena, Germany; ^4^ Department of Otorhinolaryngology, Matrix-assisted Laser Desorption/Ionization (MALDI) Imaging and Clinical Biophotonics, Jena University Hospital, Jena, Germany; ^5^ Institute of Novel and Emerging Infectious Diseases (INNT), Friedrich-Loeffler-Institut, Greifswald, Germany

**Keywords:** AAT, *Staphylococcus aureus*, virulence, NETosis, host-pathogen interaction, C-terminal Alpha-1-Antitrypsin peptides, CAAPs

## Abstract

**Background:**

The serine protease like (Spl) proteases of *Staphylococcus aureus* are a family of six proteases whose function and impact on virulence are poorly understood. Here we propose alpha-1-antitrypsin (AAT), an important immunomodulatory serine protease inhibitor as target of SplD, E and F. AAT is an acute phase protein, interacting with many proteases and crucial for prevention of excess tissue damage by neutrophil elastase during the innate immune response to infections.

**Methods:**

We used MALDI-TOF-MS to identify the cleavage site of Spl proteases within AAT’s reactive center loop (RCL) and LC-MS/MS to quantify the resulting peptide cleavage product in *in vitro* digestions of AAT and heterologous expressed proteases or culture supernatants from different *S. aureus* strains. We further confirmed proteolytic cleavage and formation of a covalent complex with Western Blots, investigated AAT’s inhibitory potential against Spls and examined the NETosis inhibitory activity of AAT-Spl-digestions.

**Results:**

SplD, E and F, but not A or B, cleave AAT in its RCL, resulting in the release of a peptide consisting of AAT’s C-terminal 36 amino acids (C36). Synthetic C36, as well as AAT-SplD/E/F-digestions exhibit NETosis inhibition. Only SplE, but not D or F, was partly inhibited by AAT, forming a covalent complex.

**Conclusion:**

We unraveled a new virulence trait of *S. aureus*, where SplD/E/F cleave and inactivate AAT while the cleavage product C36 inhibits NETosis.

## 1 Introduction

*Staphylococcus aureus* is a Gram-positive bacterium known as both asymptomatic colonizer of about 30% of the population (1, 2) and deadly pathogen. *S. aureus* is able to colonize nearly all human tissues (3) and is the leading cause of nosocomial infections involving antibiotic-resistant pathogens (4). These bacteria harbor a plethora of virulence strategies to evade the host immune system and promote colonization. These include toxins, complement pathway inhibitors and secreted proteases (5, 6). Staphylococcal proteases degrade the extracellular matrix and soluble immune effector molecules, but can also damage epithelial barriers, impair the activation of various immune cells and block complement pathways (7).

The six serine protease-like (Spl) proteases (A-F) are unique to *S. aureus*. The *spl* genes are co-transcribed (8), although different strains show varying configurations of the *spl* operon. Zdzalik et al. found that 84% of clinical isolates from various *S. aureus* infections or healthy carriers contained at least one *spl* gene, while the complete *spl* operon was only present in 31%. Overall, Spl proteases show about 30% sequence similarity to the well-known staphylococcal serine protease V8 (SspA) with their active sites being highly conserved (8). Among the Spl proteases, sequence similarity varies between 43.9% (SplD – SplA) and 94.6% (SplD – SplF) (8).

Biochemical studies using peptide libraries have provided consensus sequences for Spl protease cleavage sites (9–14), yet these sequences often do not match *in vivo* observations. Interestingly, SplB relies on a heavily restricted cleavage site motif (WELQ) and conformational change upon binding (9), while the proteolytic activity of SplC is disputed, with conflicting reports in the literature (8, 10, 14). Additionally, the pathophysiological role of Spl proteases remains poorly understood with contradictory results regarding their functions (8, 9, 15). Spl proteases are thought to selectively target specific proteins (9), yet this question is still contested (11). Until now, potential physiological targets have only been identified for SplA (mucin 16) (15) and SplB (olfactory receptor family and complement factors) (9, 16).

Here we propose alpha-1-antitrypsin (AAT) as potential physiological target of Spl proteases. AAT is an abundant plasma protein belonging to the superfamily of serpins (serine protease inhibitors) (17). It maintains the protease-antiprotease balance during inflammation, preventing excess tissue damage by released host proteases such as neutrophil elastase (17–19). As an acute phase protein, plasma levels of AAT increase 3-4 fold upon infection or inflammation (20). AAT also possesses anti-inflammatory effects independent from its protease inhibition capacity (21–23).

**Figure 1:**
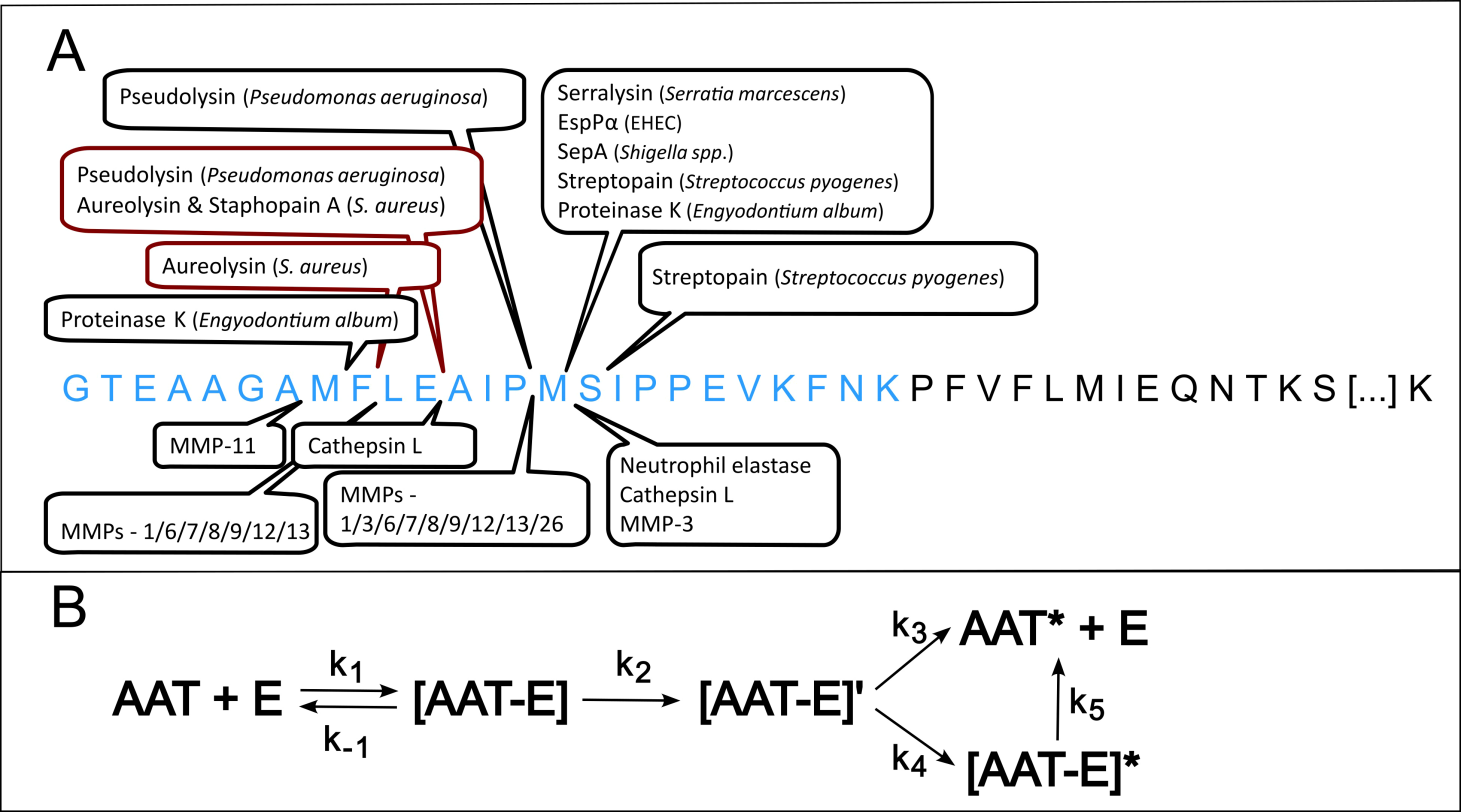
Overview of Alpha-1-Antitrypsin’s C-terminal cleavage sites and reaction mechanism **(A)** Depicts the C-terminal amino acid sequence of Alpha-1-Antitrypsin (AAT) starting at position 368, with the reactive center loop marked in blue. Cleavage sites of both exogenous as well as endogenous proteases are indicated above or below the sequence, respectively (28, 33–36, 39–49). Known cleavage sites of proteases from *S. aureus* are highlighted in dark red. **(B)** shows the reaction pathway of AAT. Upon interaction with any serine protease E, a non-covalent Michaelis complex [AAT-E] is formed. Cleavage of AAT by the protease leads to the formation of an intermediate, covalent acyl-enzyme complex [AAT-E]’, which can either be stabilized into a covalent complex with inactivated protease [AAT-E]* by AAT’s conformational change. Alternatively, the intermediate complex can also dissociate into a cleaved AAT, AAT* and the regenerated protease E. Over time, the covalent complex [AAT-E]* can slowly dissociate into AAT* and E. AAT, alpha-1-antitrypsin; k, rate constant.

Although AAT’s main target is the neutrophil elastase, it is known to interact with a multitude of both endogenous (24–29) as well as exogenous proteases (30–35) *in vitro* thus making it a prime candidate for research on novel protease substrates (see **Figure 1A**). AAT acts as a suicide inhibitor, where its C-terminal reactive center loop (RCL), extruding from the protein core, acts as a bait for proteases (36). Upon cleavage of the RCL, AAT undergoes a conformational change and the reaction follows a branched reaction pathway: AAT can either serve as substrate leaving the protease active, or as an inhibitor, covalently binding the protease (**Figure 1B**). Depending on the active site configuration of the protease and the environmental conditions (pH, temperature, etc.), either the substrate or the inhibitory pathway is favored (32, 37). The partition ratio is defined as the ratio of cleavage events to irreversible inhibition of the protease under any given conditions.

Cleavage of the RCL also releases a C-terminal peptide which we refer to as C-terminal alpha-1-antitrypsin peptide (CAAP). Depending on the protease and cleavage site, CAAPs of different lengths are generated. Therefore, the peptide pattern could serve as marker for infectious or inflammatory diseases. For example, the CAAP consisting of AAT’s C-terminal 42 amino acids (C42, formerly named CAAP48) has been proposed as sepsis marker, with its plasma concentration distinguishing infectious from non-infectious inflammation (38). In contrast, the shorter peptide C22 is present in the urine of patients with diabetic kidney disease or preeclampsia (39, 40). CAAPs might furthermore serve as prognostic markers in severe inflammatory diseases like COVID-19 or sepsis (41).

However, CAAPs are not mere byproducts of proteolytic cleavage but seem to harbor physiological functions on their own. The sepsis marker C42 for instance has been found to induce neutrophil activation, chemotaxis and IL-8 release (38). C36 exerts similar pro-inflammatory actions including activation of monocytes (42), neutrophil chemotaxis, adhesion and ROS production (35, 43), but interestingly also possesses one anti-inflammatory action by inhibiting NETosis (44). NETosis is a form of programmed cell death in neutrophils that releases neutrophil extracellular traps (NETs) to capture pathogens.

By exploring the interaction between Spl proteases and AAT, we aim to elucidate their roles in host-pathogen interaction and immune response modulation. Understanding the interplay between AAT and Spl proteases could offer novel therapeutic targets for *S. aureus* infections.

## 2 Methods

### 2.1 Heterologous expression and purification of Spl proteases

The *S. aureus* Spls A, B, D, E, and F were cloned from *S. aureus* strain USA300 (CC8-CA-MRSA), expressed as tag-free proteins in *Bacillus subtilis* 6051HGW LS8P-D, which is deficient for the proteases AprE, Bpr, Epr, Mpr, NprB, NprE, Vpr, and WprA, and purified as previously described (16, 45).

### 2.2 *S. aureus* cultivation and supernatant generation

*S. aureus* isolates were grown in tryptic soy broth (Thermo Fisher Scientific, Darmstadt, Germany) to early stationary phase with agitation at 180 rpm at 37°C. Supernatants were obtained by centrifugation of cultures at 11,000 g for 10 min at 4°C, sterilized using 0.2 µM filters (Sarstedt, Nümbrecht, Germany) and stored at -20°C until assaying.

### 2.3 *In vitro* digestion of alpha-1-antitrypsin with proteases and supernatants

Alpha-1-antitrypsin (Prolastin, Grifols Deutschland, Frankfurt, Germany) was prepared as 15 mg/mL stock solution in MS grade PBS buffer (Sigma-Aldrich, Taufkirchen, Germany) and stored in suitable aliquots at -20°C until use.

For digestion with Spl proteases, AAT stock solution was mixed with SplD, E, F and D_mut proteases in PBS buffer to achieve a 10:1 molar ratio. A negative control with buffer instead of protease was included as well. The samples were incubated at 37°C for a maximum of 24 h, sampled at indicated timepoints and stored at -80°C until measurement.

For digestion of AAT with supernatants from different *S. aureus* strains’ cultivation, 720 µL of supernatant was mixed with 80 µL of 15 mg/mL AAT stock solution (final AAT concentration 1.5 mg/mL) or PBS and incubated as described above.

### 2.4 Isoelectric focusing of AAT and AAT digestions

For AAT-IEF, the HYDRAGEL 18 A1AT ISOFOCUSING kit (Sebia GmbH, Mainz, Germany) was used. Samples were first diluted 1:10 in a provided diluent and then applied to the isofocusing gel in the HYDRASYS 2 SCAN FOCUSING device (Sebia GmbH, Mainz, Germany). The agarose gel is impregnated with ampholytes, resulting in a pH gradient of 4.2 to 4.9, which facilitates the separation of proteins according to their individual isoelectric point in the electric field.

After automated IEF, AAT was detected by immunofixation. For this purpose the gel was incubated for ten min with a peroxidase-labeled AAT-specific antiserum, whereby the peroxidase enzyme react with a substrate and generate a visible coloration. Afterwards the gel was automatically washed and dried in preparation for visual analysis. The resulting AAT profile patterns were analyzed to detect possible alterations, such as proteolytic digestion. Control samples with known phenotypes such as the MM, MZ and MS controls provided by Sebia or unprocessed commercial AAT were used.

### 2.5 MALDI-TOF-MS analysis

A saturated solution of 2,5-dihydroxybenzoic acid (DHB, Sigma-Aldrich, Taufkirchen, Germany) in MS-grade Methanol (≥99.95%, Carl Roth, Karlsruhe, Germany) containing 0.2% trifluoroacetic acid (v/v, Carl Roth, Karlsruhe, Germany) was prepared in a glass vial (VWR International GmbH, Darmstadt, Germany). 0.7 µL of each sample was spotted on a MTP384 target plate (Bruker Daltonik GmbH, US) followed by the addition of 0.7 µL of DHB matrix solution. Protein and peptide standards (Bruker Daltonik, Bremen, Germany, US) were mixed 1:1 with matrix solution before spotting (1 µL) on the target plate.

MALDI-TOF data acquisition was performed using the UltrafleXtreme mass spectrometer (Bruker Daltonik, Bremen, Germany). Measurements were carried out in positive ion reflector mode. 10.000 shots were collected across the entire sample spot in random walk with 10 shots per raster spot. The mass range incorporates for peptides 500-5500 m/z and for proteins 5-60 m/z. The Bruker peptide and protein calibration standard were used for external calibration.

For data analysis after baseline substraction and peak finding (flexAnalysis, Bruker Daltonik, Bremen, Germany) the raw spectra were exported as mzXML and further analyzed and visualized by mMass (https://github.com/xxao/mMass, GPL 3.0, Martin Strohalm).

### 2.6 LC-MS/MS analysis

For all LC-MS/MS measurements, 35 µL of sample or standard and quality control sample were mixed with 10 µL of internal standard mixture containing isotopic labelled peptides C22, C37 and C42 (0.8 µM each; sb-PEPTIDE, Saint Egréve, France), vortexed and briefly spun down. 90 µL of ice-cold methanol (≥99.95%, Carl Roth, Karlsruhe, Germany) were added to each sample, vigorously vortexed and centrifuged at 16000xg at 10°C for 10 min. Clear supernatant was transferred to 2 mL brown glass autosampler vials with 200 μL glass inserts (Wicom, Heppenheim, Germany) and subsequently subjected to analysis.

Measurements were carried out using a Shimadzu UPLC system (Duisburg, Germany) equipped with two pumps (LC-40DX3), a thermostatic autosampler (SIL-40Cx3, maintained at 10°C) and a thermostatic column compartment (CTO-40C, maintained at 45°C) coupled to a Triple Quad 5500+ mass spectrometer (AB SCIEX, Framingham, MA, USA). 10 µL of sample was injected from the autosampler and loaded on a C4 UHPLC column (Hypersil GOLD C4 1.9 µM, 2.1*100 mm; Thermo Fisher Scientific, Darmstadt, Germany). Solvent A was 0.1% formic acid (≥95%, Merck, Darmstadt, Germany) in Milli-Q water (v/v, Millipore, Brussels, Belgium) and solvent B was 0.1% formic acid in LC-grade acetonitril (v/v, ≥99.95%, Carl Roth). Chromatographic separation was achieved with a step gradient as follows: 5-30% B from 0-0.5 min., 30% B from 0.5-1 min., 30-38% B from 1-3 min., 38-41.5% B from 3-4.5 min., 41.5-85% B from 4.5-5 min., 85% B from 5-5.5 min., 85-5% B 5.5-5.7 min., 5% B from 5.7-5.8 min., 5-85% B from 5.8-6 min., 85% B from 6-6.3 min., 85-5% B from 6.3-6.6 min., 5% B from 6.6-6.7 min., 5-85% B from 6.7-6.9 min, 85% B from 6.9-7.5 min., 85-5% B from 7.5-7.7 min. and 5% B from 7.7-9 min. The flow rate was 0.35 mL/min from start to 5.5 min and 7.7-9 min, respectively, and 0.4 mL/min from 5.5-7.7 min. Detection of analytes and internal standards was carried out in multiple reaction monitoring (MRM) scan type with settings as previously published (46).

### 2.7 SDS-PAGE and western blots

1.5 mg/mL AAT was incubated with either PBS or Spl proteases (10:1 n/n) for 24 h at 37°C. 5x laemmli sample buffer was added to each sample and boiled for 5 min at 95°C. Sample containing 1, 2 or 4 µg AAT per lane were loaded on a Mini-PROTEAN precast 10% polyacrylamide gel (Bio-Rad, Germany) together with a prestained molecular weight marker (10-180 kDa, Thermo Fisher Scientific, Darmstadt, Germany) and run at constant 90 V until the desired separation was achieved.

Proteins were then wet-transferred onto a nitrocellulose membrane at 90 V for 1.5 h, before blocking with 5% bovine serum albumin (Sigma-Aldrich, Taufkirchen, Germany) in TSB buffer. Primary antibodies used were mouse anti-Nterm-AAT (NBP2-52557, NovusBiologicals, CO, USA), rabbit anti-Nterm-AAT (A99948, Antibodies.com, Sweden) and rabbit anti-Cterm-AAT (AVARP00015, Aviva Systems Biology, CA, USA). As secondary antibodies, IRDye 680LT goat anti-mouse IgG (LI-COR, Lincoln, Nebraska, USA) and IRDye 800CW goat anti-rabbit IgG (LI-COR, Lincoln, Nebraska, USA) were used. Primary antibodies were used at 1:1000 dilution and secondary antibodies at 1:15000. Membranes were dried for 1 day in the dark and analyzed with a LiCor Odyssey XF (LI-COR, Lincoln, Nebraska, USA) fluorescence imager at 700 nm and 800 nm.

### 2.8 Determination of AAT inhibition kinetics

Spl proteolytic activity was assessed by AMC [α-(4-methyl-coumaryl-7-amide)] based activity assays (47). Synthetic AMC conjugated peptide substrates spanning the Spl-consensus sequences were custom synthesized at Biomatik, Germany. The substrates used in this study were acetyl (Ac)-Arg-RYLT-AMC (SplD), Ac-LWLQ-AMC (SplE) and Ac-FYL-AMC (SplF). All peptide substrates were reconstituted in DMSO and working solutions of the substrates (25 µM) were prepared in PBS buffer at pH 7.4 for SplD and SplE and pH 8.5 for SplF.

To check whether AAT can neutralize the protease activity of Spls, Spls were either left untreated or pretreated with increasing molar ratios of AAT for 3 h at 37°C. For the substrate assay, 10 µl of native SplD or SplE (each at 2.5 µM) or SplF (25 µM) or Spls pretreated with AAT were incubated with 90 µl of the corresponding substrates to a final substrate concentration of 25 µM in a black polystyrene 96-well plate (Fluotrac, Greiner bio-one, Frickenhausen, Germany). The increase in fluorescence, indicating the release of AMC from the substrate due to the proteolytic activity of Spls, was monitored for 30 min (SplD and SplE) or 60 min (SplF) at 37°C, at excitation 380 nm and emission 455 nm using a fluorescence reader (TECAN Infinite M200, Tecan Group AG, Männedorf, Switzerland). PBS or AAT alone served as controls. All reactions were performed in duplicates.

### 2.9 NETosis assay

To assess the NETosis inhibitory potential of C36 and AAT-Spl digestions, primary human neutrophils from three healthy volunteers were pretreated with different samples and controls before NETosis stimulation with PMA. Blood was taken from healthy human volunteers after they had given their written informed consent. The blood donation protocol and use of blood for this study were approved by the Jena institutional ethics committee (Ethik-Kommission des Universitätsklinikums Jena, Permission No 2207–01/08).

In short, neutrophils were isolated from freshly drawn EDTA-blood by density-gradient isolation as described elsewhere (48). PBS + 0.1% glucose was used as assay buffer in the following procedure. The isolated neutrophils were stained for 5 min with the plasma membrane permeable DNA binding dye Sytox59 (1 µM final concentration, Thermo Fisher Scientific, Darmstadt, Germany). After washing the cells three times, 0.02*10^6^ cells were seeded per well of a transparent 96 well plate and let settle for 15 min in the dark at room temperature. C36, AAT, Spl proteases, AAT-Spl digestions or buffer were added and incubated for 15 min before addition of PMA (100 nM) or vehicle control and SytoxGreen (1 µM, Thermo Fisher Scientific, Darmstadt, Germany) mix in buffer containing 1 mM CaCl_2_ and MgCl_2_.

The cells were monitored with a Celldiscoverer 7 (CarlZeiss Microscopy, Oberkochen, Germany) for 4 h at 37°C and 5% CO_2_, taking brightfield and fluorescence (525 and 620 nm) images every 15 min. Two different areas per well were recorded.

Images were edited using Zeiss Zen 3.7 (Carl Zeiss Microscopy, Oberkochen, Germany), optimizing the settings of both fluorescence channel (Cy5 (red): black = 70, white = 786, gamma = 2.81; EGFP (green): black = 80, white = 1830, gamma = 1.73) generated equal picture quality of images over the imagining time as well as between all donors. Afterwards, the PartSeg (version 0.14.6) plug-in Trapalyzer (version 0.0.7) was used for quantitative high-throughput computational analysis of NETs in exported pictures as described elsewhere (49). NETosis was evaluated using the following filter setups: for nuclear DNA the red channel was used with median radius of 4 and a threshold of 100; NETosis was determined in the green channel with median radius of 4 and a thresholds of 20, except for PMA-stimulated samples containing SplF and AAT-SplF digestion where the threshold was set to 75 because of considerably brighter images. NETs were counted if NET pixel count was in the range of 500-70000, a NET extracellular brightness of 20-300 and NET extracellular brightness SD of 7-100. Unstimulated neutrophils were selected by a moderate size (pixel count 500-3500) and brightness (brightness 170-260; extracellular brightness 0-15, brightness gradient 0-6) and by lack of signal in the NET channel. Furthermore, Trapalyzer enables determination of various stages of NETosis, e.g. nuclear envelop rupture or plasma membrane permeabilization (for settings see **Supplementary Table 1**). All settings were adapted for a ROI quality count in the t=0 min pictures of at least 80%.

Since isolation of neutrophils from whole blood might cause damage of some cells, that were assigned as NETs, the total number of cells per image were calculated by the sum of intact cells and NETs at t=0 min. The % NETotic cells in each individual time point were calculated by the number of NETs divided by the number of total cells at t=0 min of the same picture. The mean % NETotic cells of the tow individual images of the same well were used for graphical representation.

### 2.10 Statistics

LC-MS/MS data processing was done with Analyst Software (version 1.6.2 and 1.7.1). Peak integration was reviewed individually and analyte peak areas were normalized to the peak area of their respective internal standards. Concentrations were calculated from a quadratic fit standard curve with 1/x*x weighting. All analytes with their corresponding quantification range are as previously published (46).

All data was analyzed with R version 4.3.2 (50) and graphs were plotted using the packages *gglot2* (51), and *ggpubr* (52).

## 3 Results

### 3.1 Spl D, E and F cleave alpha-1-antitrypsin *in vitro* to generate a single peptide, C36

**Figure 2:**
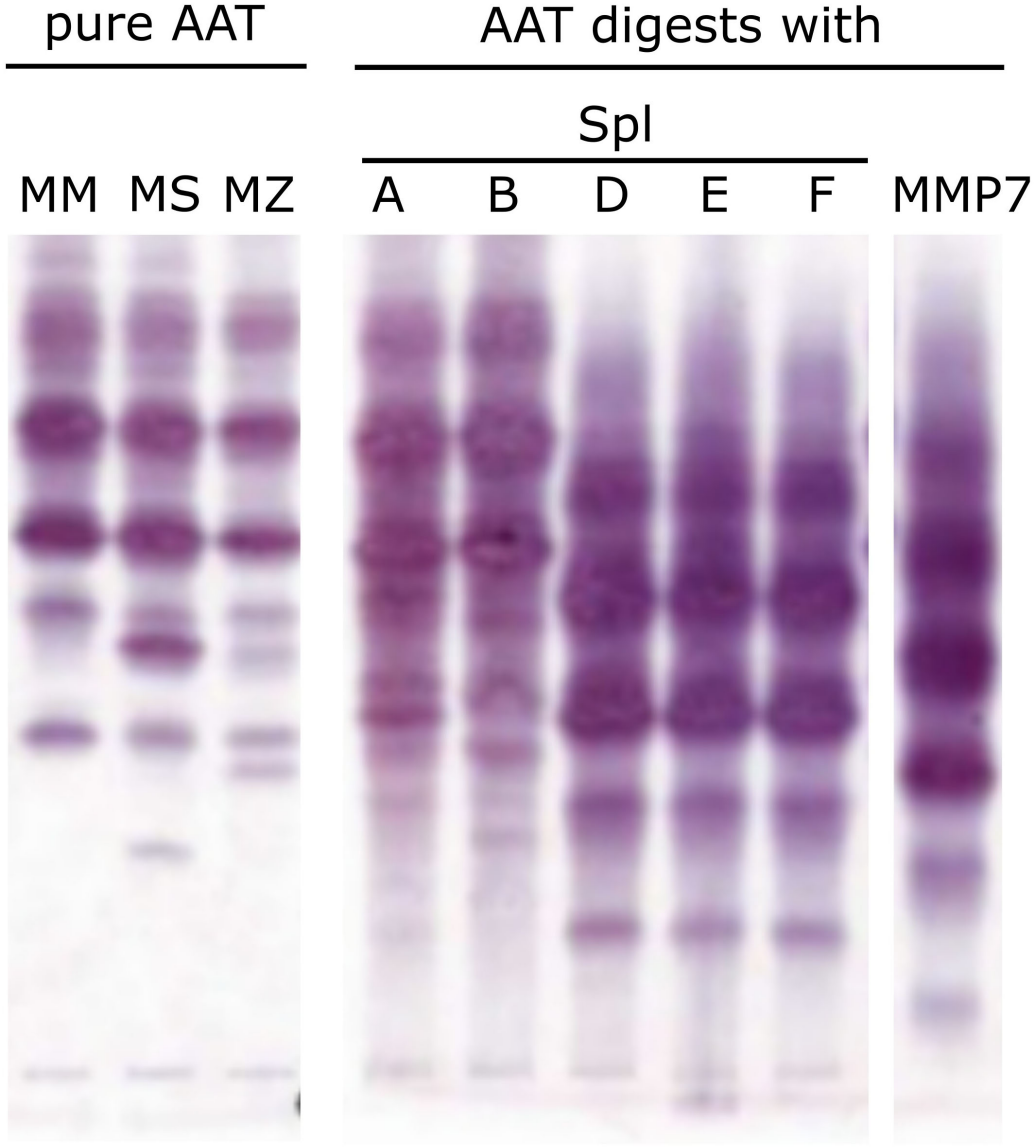
Alpha-1-Antitrypsin cleavage by Spl proteases from S. aureus Pure AAT or mixtures of AAT and indicated proteases were incubated for 3 h at 37°C before analysis by isoelectric focusing. Lanes 1-3 depict different pure AAT isoforms (normal PI*MM, pathologic PI*MS, pathologic PI*MZ genotype [4]). Upon incubation of plasma-derived AAT with SplA or B (lanes 4-5), a similar pattern can be observed. In contrast, SplD, E and F (lanes 6-8) produce a markedly different pattern, indicating cleavage of AAT. Lane 9 displays the impact of MMP7, which is known to cleave AAT at several sites, and shows a distinct pattern compared to the Spl proteases. AAT, alpha-1-antitrypsin; MMP, matrix metalloprotease.

To investigate whether any of the Spl proteases cleave AAT, we incubated AAT with recombinant Spls and analyzed the products with isoelectric focusing. While mixtures of SplA or B with AAT (**Figure 2**, lanes 4-5) showed no marked deviation from the pure AAT isoforms (lanes 1-3), SplD, E and F produced a clearly different pattern indicating cleavage of AAT. Interestingly, the three Spl proteases produced the same pattern, which is distinct from the pattern produced by MMP-7 (lane 9), a protease known to cleave AAT and generate the CAAPs C42 and C37 (29). We did not test SplC due to the known lack of protease activity.

**Figure 3:**
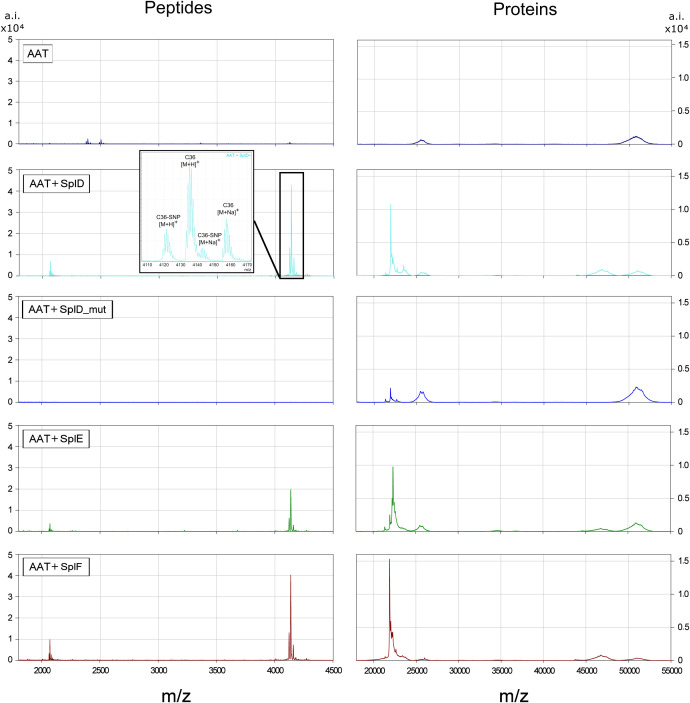
MALDI-TOF-MS spectra of AAT and AAT-Spl digestions Spectra display the peptide mass range (1700-4500 m/z) on the left and protein mass range on the right (17000-55000 m/z) of the average of n=3 independent experiments. The inlet shows a zoomed-in portion of the respective spectra to better distinguish the signals. AAT or AAT plus respective Spl protease was incubated in PBS at 37°C for 21 h before sample preparation and measurements as described in Materials and Methods. Single charged native or digested AAT can be seen around 52000 or 47000 m/z, respectively. Spl proteases are visible at approximately 22500 m/z. While SplD_mut did not show any digestion of AAT, SplD, E and F all cleaved AAT at the same site. AAT, alpha-1-antitrypsin.

To identify the exact cleavage sites of SplD, E and F, similar digestions with AAT were further investigated using untargeted MALDI-TOF-MS analysis. As seen in **Figure 3**, pure AAT shows single and double charged peaks at around 52.000 and 26.000 m/z, respectively. Only minor signals were detected in the peptide mass range. These are explained by the fact that AAT was isolated from plasma where small amounts of CAAPs are physiologically present and adhered to AAT. Addition of heterologously expressed and purified SplD, E or F leads to proteolytic cleavage of AAT, marked by a decrease in native AAT at 52.000 m/z and the appearance of a slightly smaller AAT species at around 47.000 m/z. At the same time, prominent signals in the peptide mass range appeared, representing a single and double charged peptide at 4133.3159 and 2067.4203 m/z, respectively, as well as sodium adducts (+11.99 m/z) and SNP variants (-14.03 m/z) thereof. The AAT gene harbors a frequent single nucleotide polymorphism (SNP) rs1303, leading to a Glu > Asp substitution at Glu386 (19^th^ AA from the C-terminus). The occurrence of signals corresponding to the SNP variants therefore localizes the corresponding peptides within the C-terminal part of AAT. Matching the observed mass against all possible peptides containing the C-terminal reactive center loop and the SNP site, identified the peptide as C36 (theoretical monoisotopic mass (M+H)^+^: 4133.2330, observed: 4133.3159, Δppm = 20.0).

We also tested a SplD mutant (SplD_mut, Ser156Ala) lacking protease activity as a negative control. As expected, SplD_mut did not cleave AAT and did not produce C36 or other CAAPs.

**Figure 4:**
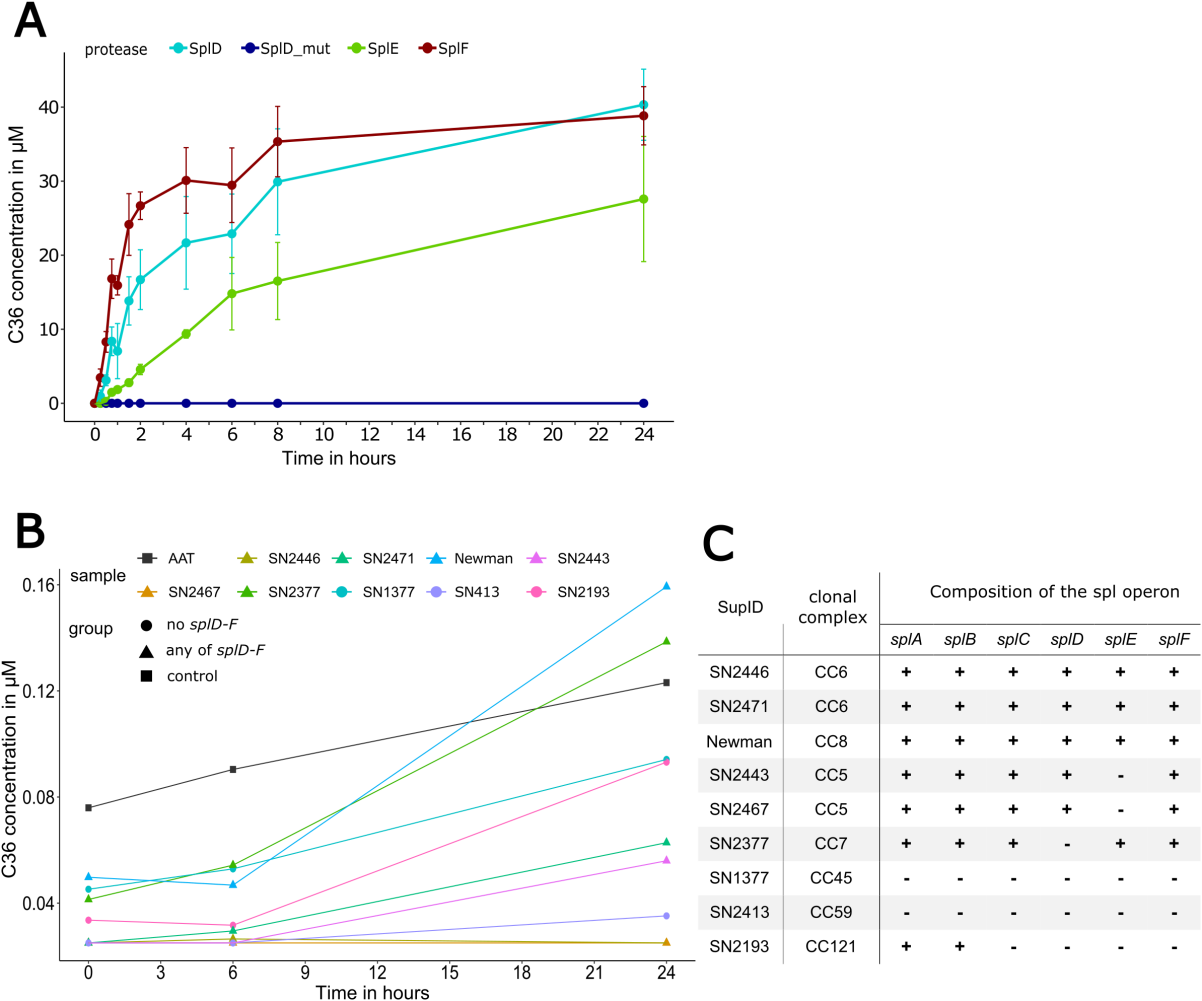
Proteolytic cleavage of AAT by Spl proteases or *S. aureus* culture supernatants 1.5 mg/mL AAT was incubated with either purified Spl proteases D, D_mut, E and F at 1:10 molar ratio **(A)** or with supernatants from different *S. aureus* strains **(B)** harboring different spl operon compositions **(C)**. In **(A)**, values represent the mean ± standard deviation of three independent replicates. Concentration levels in **(B)** are normalized to the total protein amount of the supernatants and represent the mean of two independent experiments. Strains in **(B)** are grouped by either containing any combination of splD, splE and splF or none thereof. AAT, alpha-1-antitrypsin.

After identification of C36 as cleavage product of AAT, we set out to determine the amount and kinetics of C36 being generated using a quantitative LC-MS/MS method. Incubating AAT with Spl proteases at a 10:1 molar ratio and subsequent sampling over 24 h revealed no generation of C36 by SplD_mut, in accordance with IEF and MALDI-TOF-MS results (**Figure 4A**). In contrast, both SplD and F displayed typical enzymatic kinetics and the resulting C36 concentrations follow a saturation curve. After 24 h, C36 concentrations resemble initial AAT concentrations, indicating a complete turnover of AAT. Intriguingly, SplE displays a different pattern with markedly slower C36 generation and reduced maximal C36 concentration indicating an irreversible inhibition of SplE by AAT.

### 3.2 C36 is also generated by various *S. aureus* culture supernatants

In a second set of experiments, AAT was incubated with the culture supernatants obtained from nine different *S. aureus* strains instead of single proteases. These supernatants contain a variety of *S. aureus* secreted proteases and therefore more closely resemble an *in vivo* environment. The table in **Figure 4C** depicts the *S. aureus* strains’ composition of the *spl* operon. We quantified CAAPs in pure AAT preparation and in the mixtures of bacterial culture supernatants and AAT using our LC-MS/MS method. As expected, no traces of C36 or other CAAPs could be detected in any of the pure *S. aureus* supernatants (data not shown). In contrast, minor amounts of C36, C40, C42 and C43 were present in the pure AAT preparation, which increased slightly over time, as CAAPs bound to AAT slowly dissociate and become measurable.

As shown in **Figure 4B**, only the supernatants of the strains SN2471 (splA-F) and SN2377 (splA-C,E,F) displayed protease activity generating more C36 than the AAT control, and only after 24 h. Notably, the presence of Spl proteases was only assessed on a genomic level (**Figure 4C**), and the protein expression levels of Spl proteases may vary greatly between strains. Interestingly, some bacterial supernatants contained additional protease activity generating C40 and C42, known cleavage products of Aureolysin and Staphopain A, respectively (**Supplementary Figure 1**), further supporting the hypothesis of different protease expression levels between strains (33, 53).

### 3.3 AAT inhibits SplE, but not SplD or SplF

**Figure 5:**
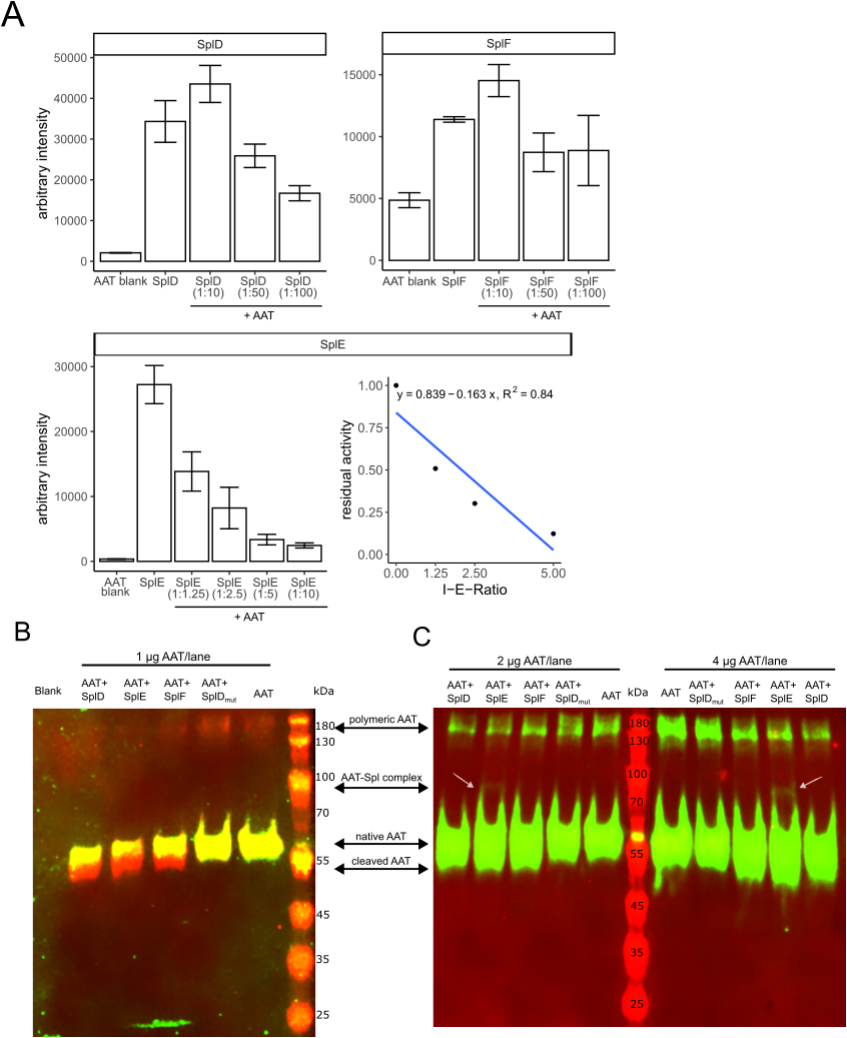
Effect of AAT on Spl protease activity **(A)** Native Spls or Spls pretreated with indicated molar ratios of AAT were incubated with AMC-conjugated substrates as described in the methods section. Bar graphs show mean fluorescence intensities ± standard deviation after 30 min (for SplD and SplE) or 60 min (SplF) reaction time of two or three experiments performed in duplicates. For SplE, the residual activity was plotted against the inhibitor-enzyme ratio (I-E-Ratio) to determine the partition ratio (x-axis intercept). **(B, C)** display Western Blots of AAT or AAT-Spl digestions with indicated protein amounts per lane. In **(B)**, a mix of two sets of primary and secondary antibodies was used to detect the C-terminus of AAT as green and the N-terminus as red fluorescence. As the C-terminus is cleaved off by SplD-F, the cleaved AAT only contains the native N-terminus and is therefore only detected in the red channel. In **(C)**, only one set of antibodies was used to detect the N-terminus of AAT. Native and cleaved AAT therefore are both detected in the green channel. AAT, alpha-1-antitrypsin; AMC, 7-Amino-4-methylcoumarin.

To further investigate the interaction of AAT with the Spl proteases, we used a chromogenic assay with small synthetic peptide substrates to assess the inhibitory capacity of AAT towards SplD-F. As seen in **Figure 5A**, AAT exclusively inhibits SplE at proposed physiological molar ratios (1:10 and below), while SplD and SplF were only slightly inhibited at excess amounts of AAT (1:50 and 1:100 molar ratios). Notably, the inhibition of SplE by AAT was concentration dependent, and by plotting the residual activity against the inhibitor-enzyme ratio, we determined the partition ratio to be 5.15. Therefore, about 5 moles of AAT were necessary to inhibit 1 mole of SplE.

These findings were further strengthened by Western Blot analysis of AAT-Spl digestions. In accordance with the MALDI-TOF data (**Figure 3**) and the C36 levels (**Figure 4A**) that were generated, SplD-F but not SplD_mut cleaved AAT (**Figures 5B, C**). Notably, when loading larger amounts of digest per lane, we observed a slight protein band at approximately 80 kDa which was only present in the AAT-SplE digests. The size of this band fits to the sum of AAT (51 kDa) and SplE (22 kDa), indicating the formation of a covalent complex between the two proteins, albeit in small amounts. Since AAT’s protease inhibition mechanism is facilitated by formation of a covalent complex, this finding matches the observation that only SplE is inhibited by AAT (**Figure 5A**).

### 3.4 Both synthetic C36 and AAT + Spl D/E/F digestions inhibit NETosis

Lastly, we investigated possible patho-physiological effects of AAT digestion and C36 generation by SplD-F. As C36 is known to inhibit NETosis, and NETosis is an important innate immune effector mechanism to contain infections, we asked whether AAT digested by SplD, E or F can also interfere with NETosis.

**Figure 6:**
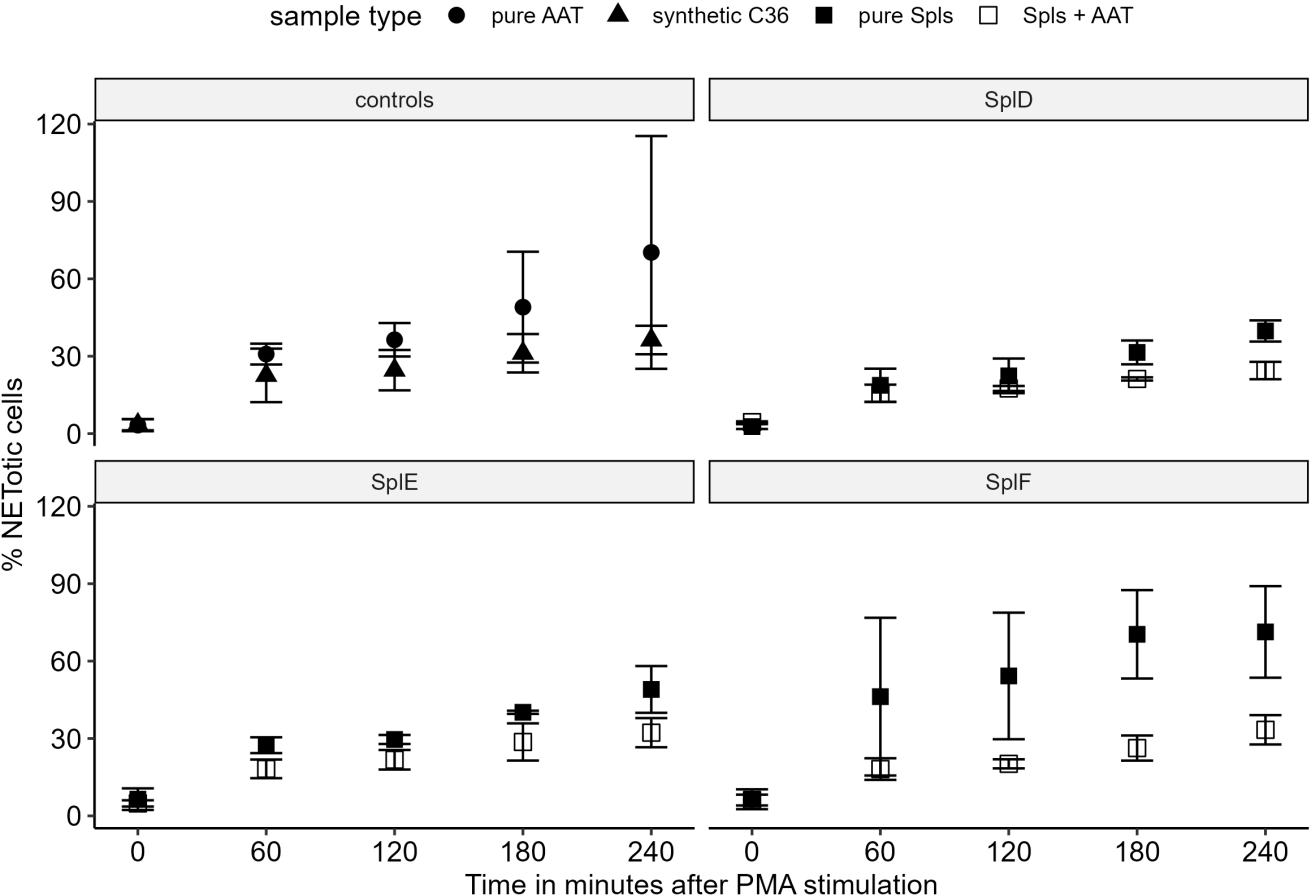
NETosis inhibition by AAT and AAT-Spl digestions Isolated neutrophils from healthy donors were preincubated with respective proteins or peptides with subsequent NETosis induction by PMA. Cells were monitored for 4 h and the percentage of NETotic cells was determined by automated image processing. The percentage of NETotic cells over time is displayed as mean +/- standard deviation of three biological replicates with two technical replicates each. Comparing synthetic C36 to AAT or AAT-Spl digestions to the respective pure proteases, a moderate to strong NETosis inhibition can be observed. AAT, alpha-1-antitrypsin; PMA, Phorbol 12-myristate 13-acetate.

When isolated neutrophils from healthy donors were exposed to synthetic C36, AAT, pure Spl proteases or digestions of AAT with Spls, only a moderate degree of NETosis was induced (**Supplementary Figure 2**). Subsequent stimulation with PMA markedly increased the NETosis rate, up to 70% NETotic cells (**Figure 6**). Compared with AAT preincubation, C36 treatment attenuated the PMA-induced NETosis by approximately 50%. A similar but slightly weaker inhibition was observed when neutrophils were treated with protease-AAT digestions compared to protease alone. Microscopic pictures of live cell imaging are available in **Supplementary Presentation 1**.

## 4 Discussion

In this work, we showed for the first time that SplD, E and F cleave the reactive center loop of AAT *in vitro* to produce the C-terminal peptide C36. Interestingly, those three proteases have the highest sequence similarity among all Spl proteases. The shared cleavage site is therefore not unexpected. SplB, in contrast, did not cleave AAT, in accordance with previous findings (9).

The cleavage of AAT by SplD, E and F is remarkable because of two aspects. First, these Spl proteases utilize the same cleavage site as AAT’s main target, the neutrophil elastase. Secondly, Spl proteases were assumed to be specialized proteases covering distinct substrates (9), as their biochemically established consensus cleavage sites vary (11–14). In our experiments however, all three proteases cleaved AAT at the same site. Notably, only one cleavage occurred even after prolonged incubation times, highlighting the importance of this specific cleavage site and ruling out unspecific proteolytic degradation of AAT by Spls.

We also observed the generation of C36 by extracellular proteins released by two *S. aureus* strains. While under these conditions the formation of C36 cannot be attributed to a specific protease, no other *S. aureus* proteases have so far been shown to cleave AAT at the C36 cleavage site, proposing the Spl proteases as the originators. However, we did not observe a clear correlation between the amount of C36 and the number or combination of spl genes present in the different strains. This is probably due to large differences in gene expression between *S. aureus* isolates, resulting in highly variable exoproteomes (54, 55). Supporting this hypothesis, also the amounts of C40 and C42 generated from AAT by incubation with *S. aureus* culture supernatants varied considerably between the different bacterial strains, despite their similar genetic background (**Supplementary Figure 1**). Overall, we detected considerably lower amounts of C36 when incubating AAT with *S. aureus* culture supernatants compared to purified Spl proteases. This could be explained by lower concentrations of Spl proteases present under these circumstances. While we did not quantify Spl levels in the supernatants used in our study, there are first investigations determining the SplB concentration in stationary TSB cultures as about 0.5 µg/mL (Personal communication Kristin Surmann, Interfaculty Institute for Genetics and Functional Genomics, University of Greifswald, Germany). Assuming that the other Spl proteases have a similar expression, the concentration of Spl proteases in the AAT-supernatant digestions would be approximately 15 times lower than in the AAT-protease digestions, explaining lower C36 concentrations. However, these are only rough estimates, especially in the context of apparent variations in gene expression between different strains. *S. aureus* gene expression might also change drastically upon interaction with host factors during an infection compared to axenic cultures.

Given the well documented appearance of C36 in clinical settings such as severe infections (46, 56) and the multiple effects of C36 on immune cells (25, 26, 42, 43, 57, 58), the generation of C36 by SplD, E and F may have pathophysiological implications during *S. aureus* infections.

In support, our experiments showed that preincubation with digestions of AAT and SplD, E or F strongly reduces PMA-induced NETosis in neutrophils from healthy blood donors compared to preincubation with pure proteases. The effect size was similar to that of synthetic C36. These results suggest that *S. aureus’* SplD, SplE and SplF function as virulence factors by generating C36, which reduces NETosis and thereby promotes *S. aureus* immune evasion and dissemination.

On the other hand, C36 also has been shown to entail activating effects on immune cells, such as increasing ROS production, neutrophil chemotaxis and adhesion as well as cytokine production. These pro-inflammatory favor *S. aureus* detection and elimination by the innate immune response. The overall effect of C36 on *S. aureus* immune control vs. immune evasion will probably depend on the context.

When evaluating the overall effect of AAT cleavage and C36 generation, the partial inhibition of Spl proteases in the process needs to be considered as well. Using small synthetic peptides to measure protease activity, we found that only SplE was inhibited by AAT, but not SplD or F. SplD and F share a sequence similarity of 94.6%, while SplE is only 64.4 or 64.8% similar to D and F, respectively (7). As the active site configuration of the protease is one of the determining factors in whether a protease is covalently bound by AAT, it is not surprising that SplD and F behave similar, yet distinct to SplE. The inhibition of SplE was concentration dependent, and an approximately 5-fold molar excess of AAT was needed to completely abolish protease activity. This is best explained by a branched reaction pathway for SplE-mediated cleavage of AAT with a partition ratio of 5, meaning that only 1 of 5 cleavage events leads to an irreversible inhibition of the protease. This highlights that AAT functions more as a substrate and less as an inhibitor in this interaction. Other proteases have also been reported to follow a similar branched reaction pathway when cleaving AAT, e.g. matriptase (59). For comparison, AAT’s main target of inhibition, the neutrophil elastase, has a partition ratio of close to 1 indicating a rapid and near complete inhibition of elastase upon association with AAT (32).

Furthermore, it is not only the protease that is inactivated upon cleavage, but also AAT. As a suicide inhibitor, AAT is no longer active after cleavage of its reactive center loop, regardless of whether the protease is irreversibly trapped or not (32). This means that at a partition ratio of about 5, 5 moles of AAT are inactivated for each mole of SplE protease. As one of the major endogenous protease inhibitors, inactivation of AAT shifts the local protease-antiprotease balance and may not only affect host proteostasis but also prevent inactivation of other *S. aureus* proteases.

Similar observations regarding AAT inactivation enhancing infection severity have been made by Gogol et al. investigating *Candida albicans* (31). The authors found that *C. albicans*’ secreted aspartic proteases cleave AAT in its C-terminal region at several sites different from the C36 cleavage site reported here. Protease-mediated inactivation of AAT by Saps resulted in deregulation of neutrophil elastase, damage to epithelial and endothelial cells, and increased colonization of these compromised cells by *C. albicans*.

However, there are limitations in our experimental setup. First, all experiments were conducted *in vitro* and we did not study *S. aureus* infections *in vivo*. Nevertheless, we aimed to imitate the *in vivo* environment as closely as possible. The AAT concentrations used in our experiments are equivalent to human plasma concentrations. The AAT to protease molar ratios were set to 10:1, resembling AAT abundance at local sites of infection. Together with our use of *S. aureus*’ secretome, we sought to recreate the *in vivo* conditions of infection as closely as possible.

Second, the NETosis inhibition was investigated using recombinant proteases, synthetic C36 and isolated AAT, rather than exposing the neutrophils to live *S. aureus*. This was necessary to uncover the molecular function of the Spls. Further experiments could study *S. aureus* induction and survival of NETosis under different AAT or C36 supplementation. In addition, the multiple other functions of C36 were not studied here. Therefore, the assessment of the overall effects of C36 generation by Spl proteases on *S. aureus*-host-interaction requires further study.

Our findings uncovered a new virulence trait of *S. aureus*: cleavage and inactivation of the human protease inhibitor AAT, production of C36, and inhibition of NETosis. The inactivation of AAT with only moderate (SplE) or no loss of protease activity (SplD and SplF) could shift the local protease-antiprotease balance possibly favoring *S. aureus* colonization. The bacterial proteases might be a future target for an anti-virulence treatment strategy similar to the use of monoclonal antibodies to neutralize *S. aureus*’ pore forming toxins or to sensitize *S. aureus* to ROS by inhibiting the antioxidant staphyloxanthin (60). In the case of Spls, small molecular weight protease inhibitors might be a suitable option. Alternatively, AAT supplementation may compensate for the loss of activity by Spl cleavage. Further *in vivo* studies are needed to confirm and expand our novel findings of the interaction between Spl proteases and alpha-1-antitrypsin.

## Data Availability

The raw data supporting the conclusions of this article will be made available by the authors, without undue reservation.
