# Supplementary figures and images for: Alpha-1-antitrypsin as novel substrate for S. aureus’ Spl proteases – implications for virulence
Source: Front Immunol. 2024 Nov 19;15:1481181. doi: 10.3389/fimmu.2024.1481181 (PMC11611844; doi:10.3389/fimmu.2024.1481181)

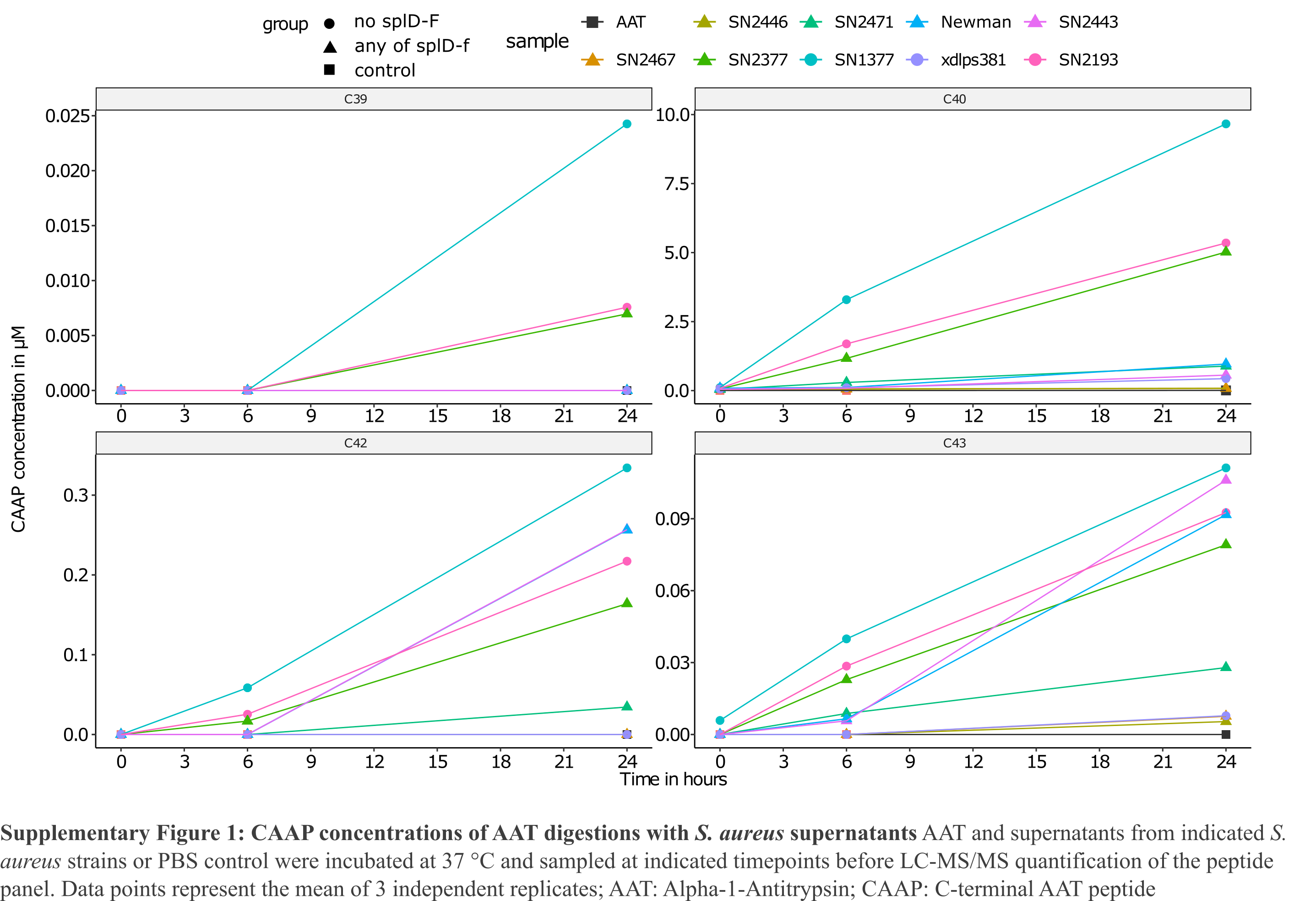

Supplement: Supplementary file 1 [file Image1.tiff]

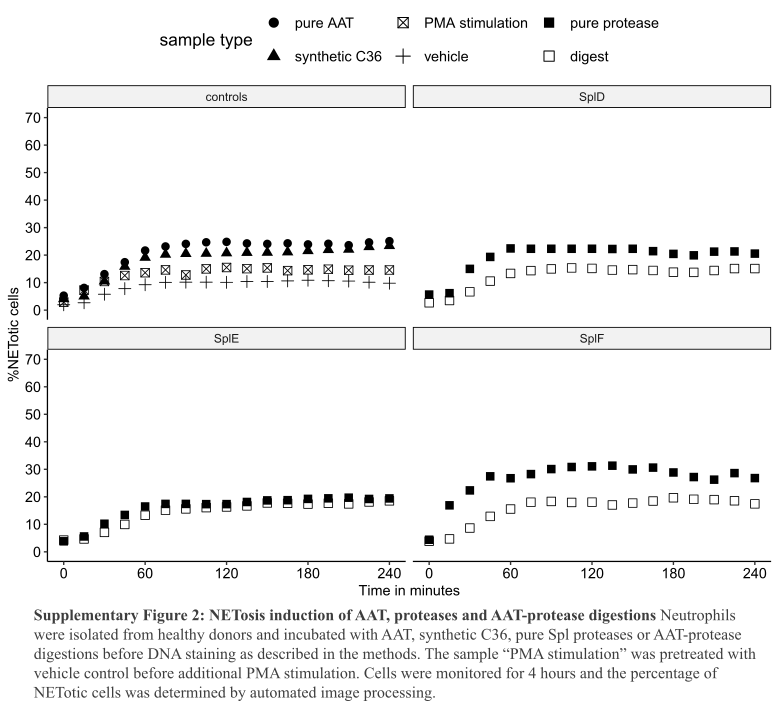

Supplement: Supplementary file 2 [file Image2.tiff]
