# Supplementary material for: Alpha-1-antitrypsin as novel substrate for S. aureus’ Spl proteases – implications for virulence
Source: Front Immunol. 2024 Nov 19;15:1481181. doi: 10.3389/fimmu.2024.1481181 (PMC11611844; doi:10.3389/fimmu.2024.1481181)
Supplement: Supplementary file 3 [file Presentation1.pptx]

## Slide 1
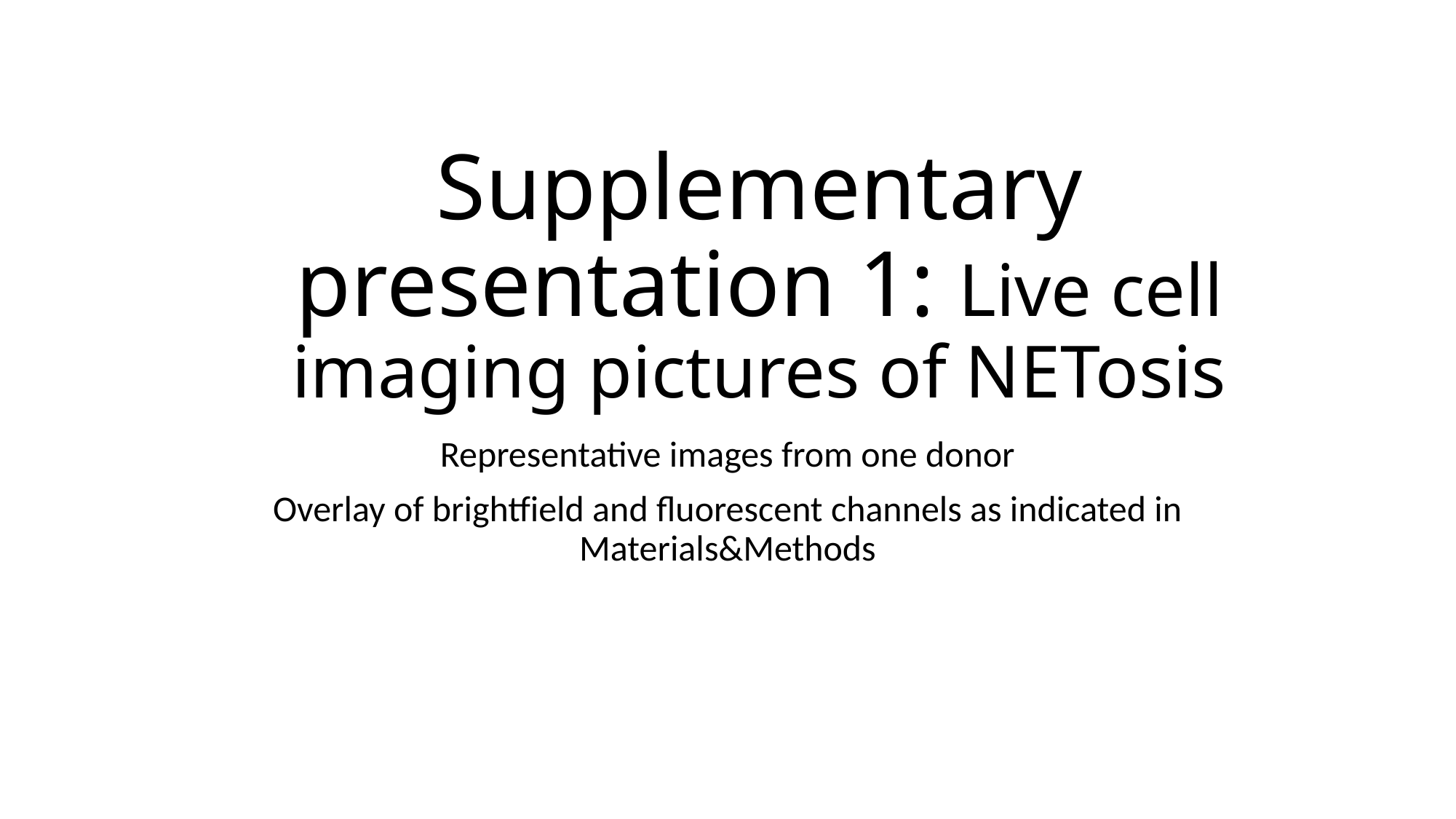

# Supplementary presentation 1: Live cell imaging pictures of NETosis
Representative images from one donor
Overlay of brightfield and fluorescent channels as indicated in Materials&Methods

## Slide 2
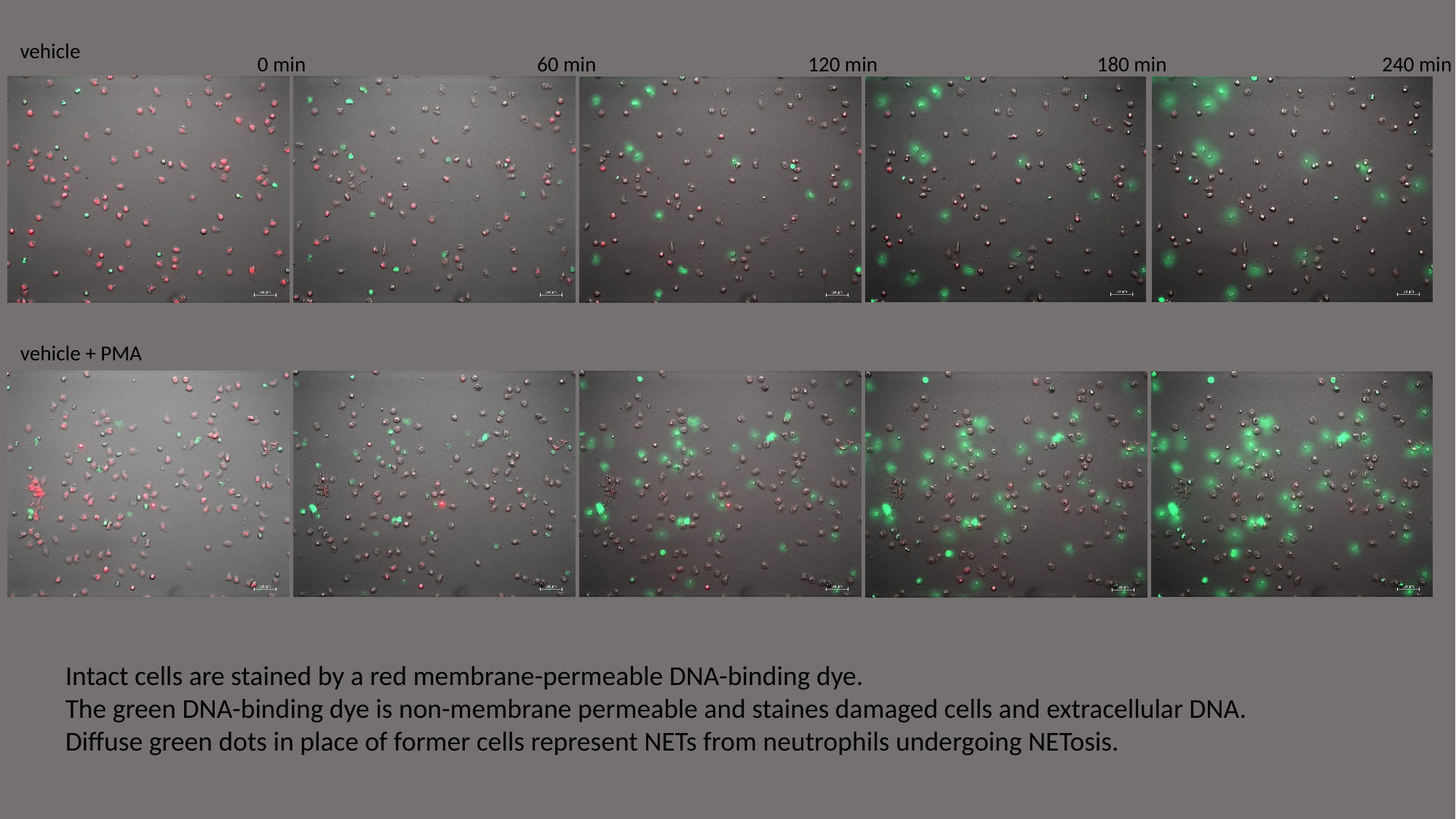

vehicle
0 min		 60 min	 	 120 min		 180 min		 240 min
vehicle + PMA
Intact cells are stained by a red membrane-permeable DNA-binding dye.
The green DNA-binding dye is non-membrane permeable and staines damaged cells and extracellular DNA. Diffuse green dots in place of former cells represent NETs from neutrophils undergoing NETosis.

## Slide 3
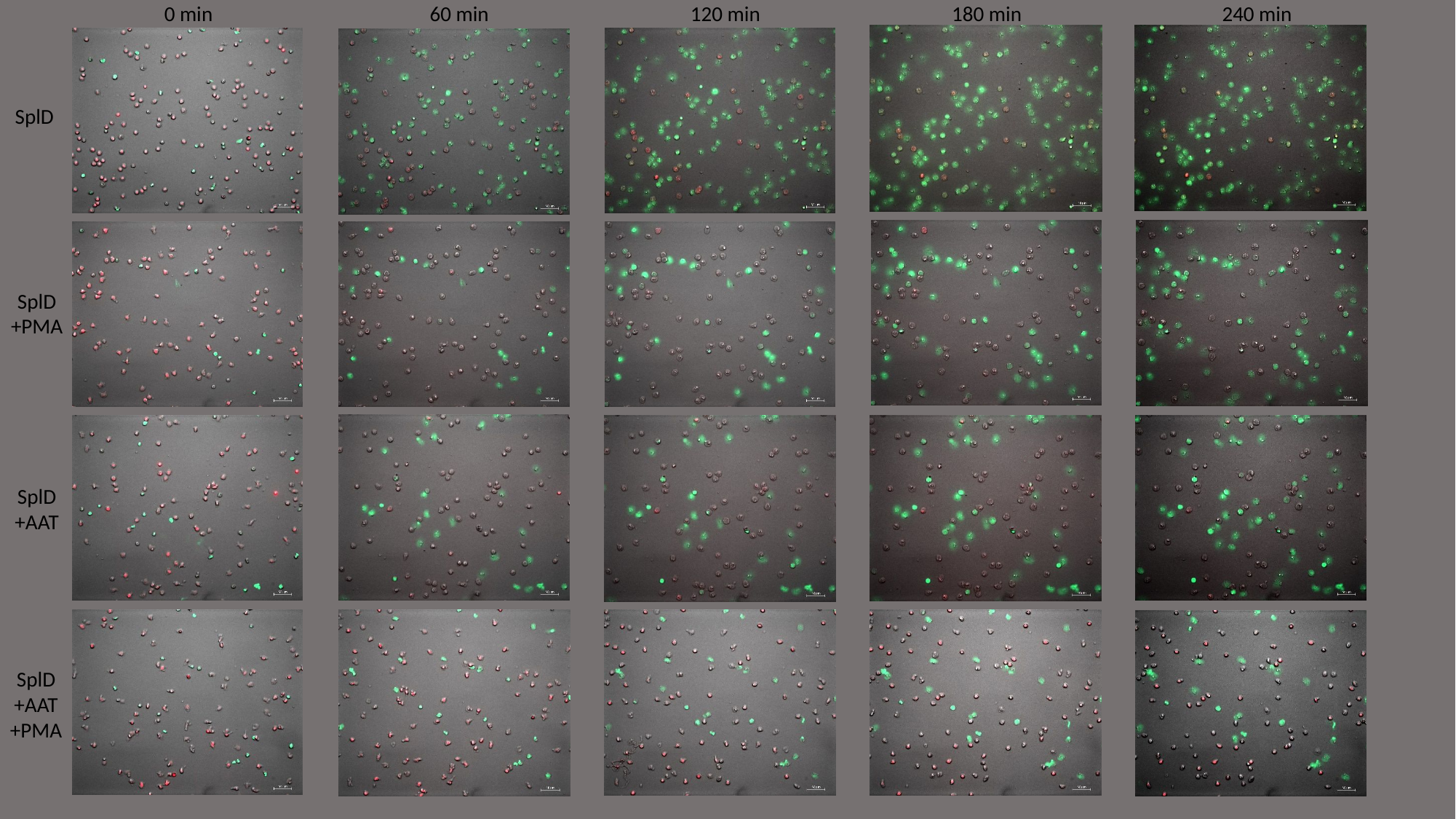

0 min		 60 min		 120 min		 180 min		 240 min
SplD
SplD +PMA
SplD +AAT
SplD +AAT+PMA

## Slide 4
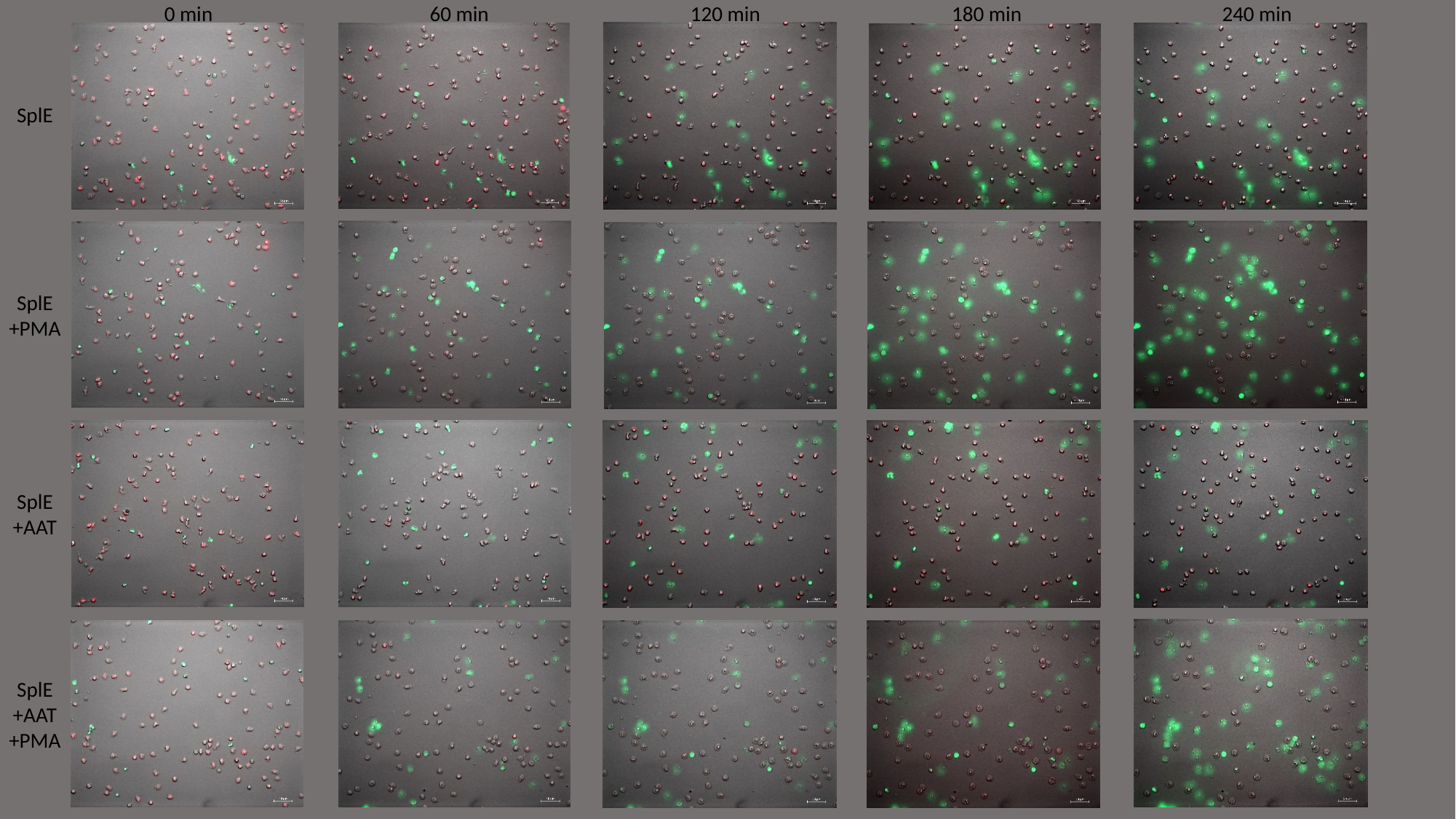

0 min		 60 min		 120 min		 180 min		 240 min
SplE
SplE +PMA
SplE +AAT
SplE +AAT+PMA

## Slide 5
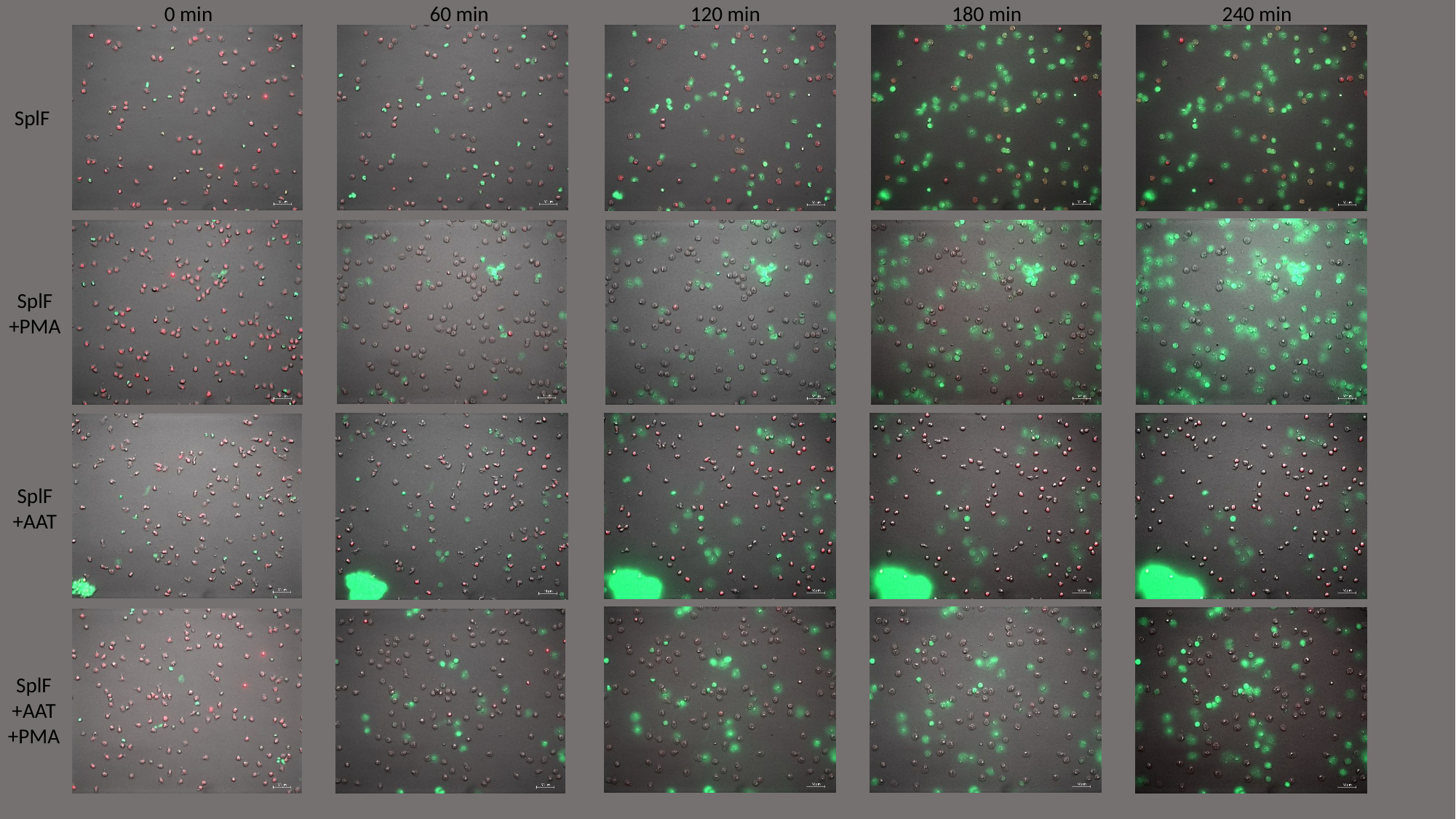

0 min		 60 min		 120 min		 180 min		 240 min
SplF
SplF +PMA
SplF +AAT
SplF +AAT+PMA

## Slide 6
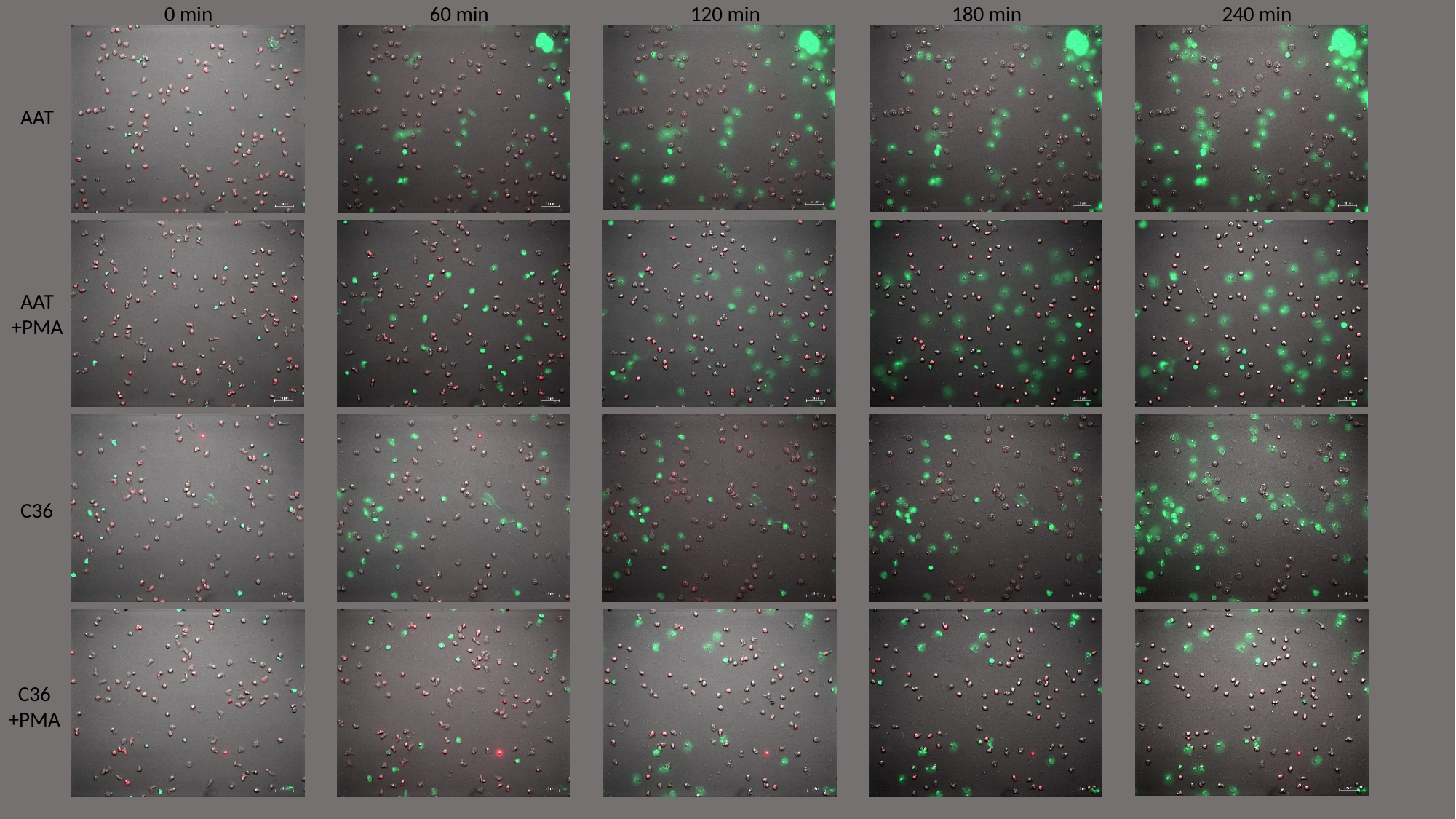

0 min		 60 min		 120 min		 180 min		 240 min
AAT
AAT +PMA
C36
C36 +PMA
