# Supplementary material for: Alpha-1-antitrypsin as novel substrate for S. aureus’ Spl proteases – implications for virulence
Source: Front Immunol. 2024 Nov 19;15:1481181. doi: 10.3389/fimmu.2024.1481181 (PMC11611844; doi:10.3389/fimmu.2024.1481181)
Supplement: Supplementary file 4 [file Table1.docx]

Supplementary Material

**Supplementary Table 1: Settings for Trapalyzer analysis of NETosis in fluorescence images**

|  | Intact  neutrophil | rounded nucleus | nuclear envelop rupture | plasma membrane permeabilisation |
| --- | --- | --- | --- | --- |
| pixel count | 500.00 - 35,000.00 | 0.00 - 1.00 | 100.00 - 12,000.00 | 500.00 - 1,000.00 |
| brightness | 150.00 - 260.00 | 0.00 - 1.00 | 20.00 – 120.00 | 5.00 - 340.00 |
| extracellular brightness | 0.00 - 15.00 | 0.00 - 1.00 | 0.00 - 20.00 | 20.00 - 80.00 |
| brightness gradient | 0.00 - 6.00 | 0.00 – 0.01 | 0.05 - 15.00 | 0.25 - 1.50 |
